# Supplementary material for: Thermal cues drive plasticity of desiccation resistance in montane salamanders with implications for climate change
Source: Nat Commun. 2019 Sep 9;10:4091. doi: 10.1038/s41467-019-11990-4 (PMC6733842; doi:10.1038/s41467-019-11990-4)
Supplement: Supplementary file 1 — Supplementary Information [file 41467_2019_11990_MOESM1_ESM.pdf]

Thermal cues drive plasticity of desiccation resistance in montane salamanders with implications for climate change

Riddell et al.

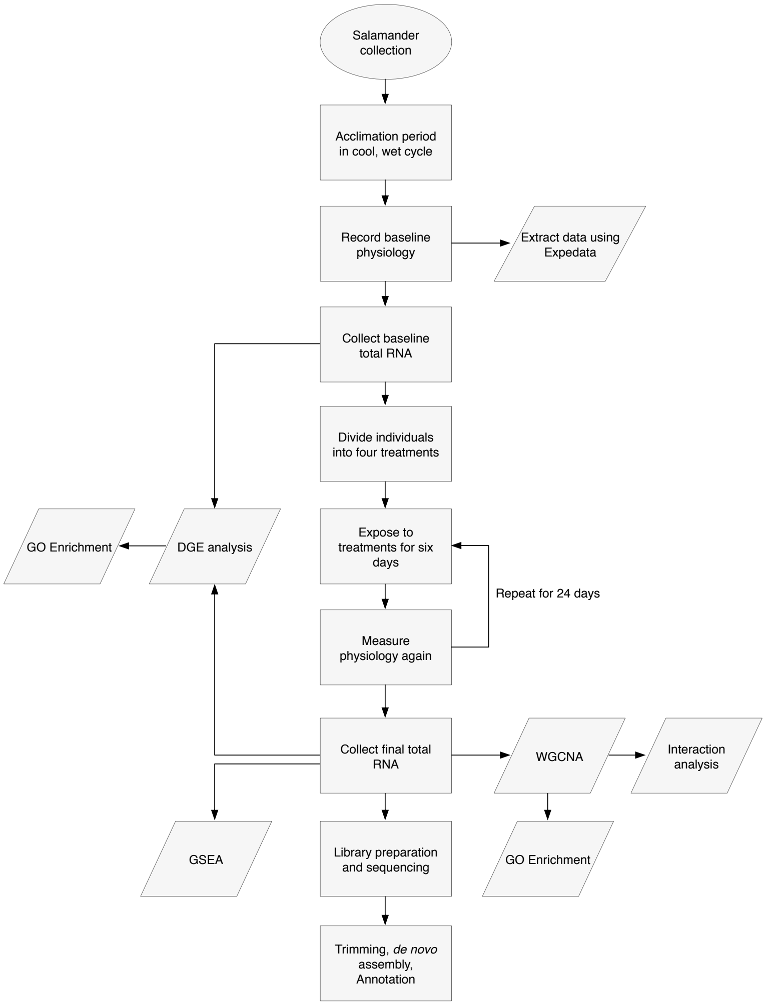

Supplementary Figure 1. Flow chart of the experiment and transcriptional analyses providing a brief overview of the experiment for clarity. Circle indicates the beginning of the experiment, rectangles are procedures in the laboratory, and rhombuses are computational analyses.

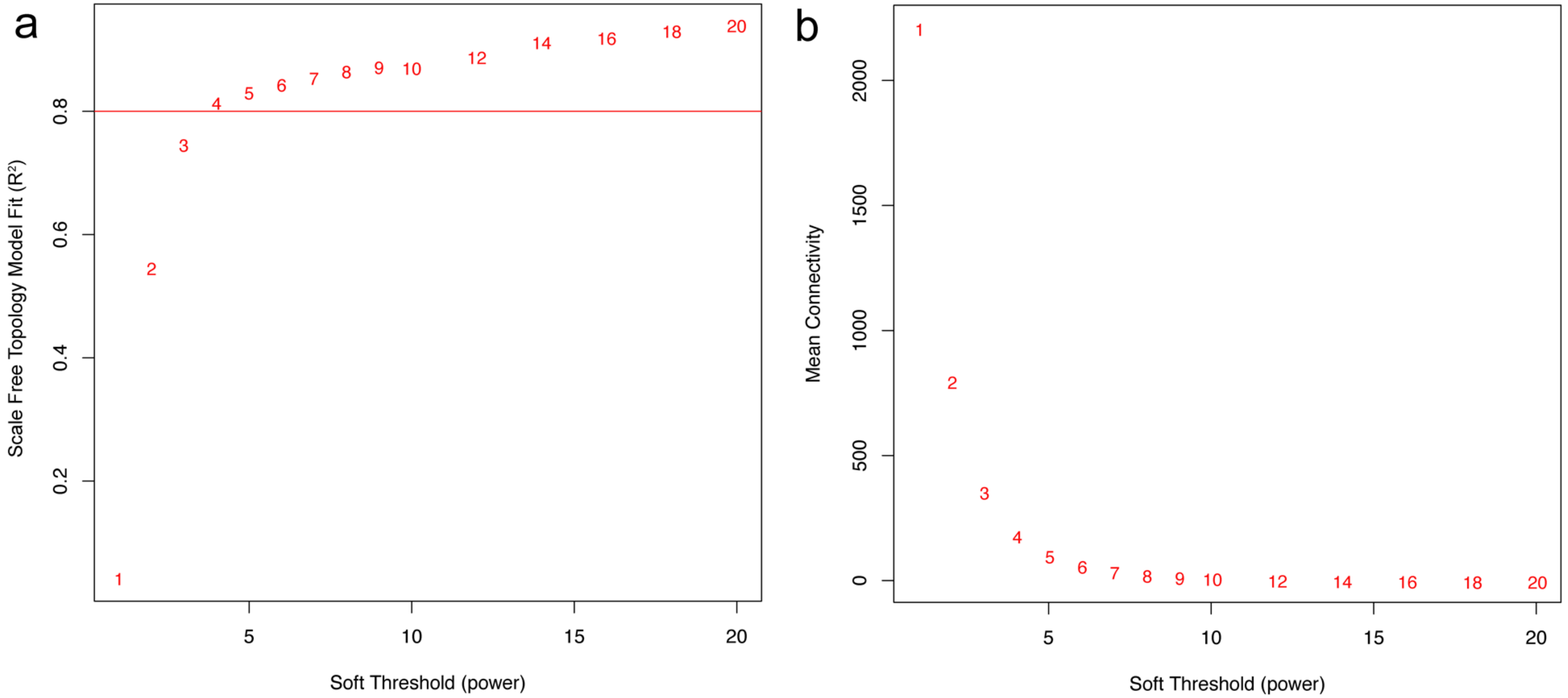

Supplementary Figure 2. The scale free topology (a) and mean connectivity (b) used to identify the soft threshold for the WGCNA. The horizontal red line indicates the 80% R^2 threshold used to identify the threshold value of 4 used in our study.

Supplementary Table 1. Statistics for non-linear mixed effects model of the relationship between temperature and VPD (top panel) and temperature and the variation in VPD (bottom panel). Comparisons of the parameter estimates with their standard error indicates that all parameters were different from zero.

**VPD model**			
$\sim k + a^{(b \times \text{temperature})}$			
**Random effects**	***Variance***	***Std. Dev.***	
k | Year/Coordinate ID	1.37×10^{-2}	1.17×10^{-1}	
a | Year/Coordinate ID	1.86×10^{-6}	1.36×10^{-3}	
**Fixed effects**	***Estimate***	***Std. Error***	***t-value***
k	1.33×10^{-2}	4.22×10^{-3}	3.15
a	1.03×10^{-3}	1.85×10^{-4}	5.60
b	2.68×10^{-1}	1.75×10^{-3}	152.75

**$\sigma_{\bar{x}}$ of VPD model**			
$\sim k + a^{(b \times \text{temperature})}$			
**Random effects**	***Variance***	***Std. Dev.***	
b | Year/Coordinate ID	2.67×10^{-3}	5.17×10^{-2}	
**Fixed effects**	***Estimate***	***Std. Error***	***t-value***
k	1.26×10^{-2}	1.93×10^{-3}	6.52
a	6.09×10^{-7}	2.79×10^{-7}	2.18
b	5.90×10^{-1}	3.44×10^{-2}	17.11

Supplementary Table 2. GO term enrichment for differentially expressed genes down-regulated in response to warm temperatures.

Category	*p*-value	Term	Ontology
GO:0048536	7.74E-05	spleen development	BP
GO:0006397	9.93E-05	mRNA processing	BP
GO:0048863	1.18E-04	stem cell differentiation	BP
GO:0000381	2.44E-04	regulation of alternative mRNA splicing, via spliceosome	BP
GO:0048538	5.02E-04	thymus development	BP
GO:0045814	5.16E-04	negative regulation of gene expression, epigenetic	BP
GO:0000245	6.64E-03	spliceosomal complex assembly	BP
GO:0030514	6.75E-03	negative regulation of BMP signaling pathway	BP
GO:0001889	1.03E-02	liver development	BP
GO:0045930	1.14E-02	negative regulation of mitotic cell cycle	BP
GO:0000122	1.16E-02	negative regulation of RNA polymerase II promoter	BP
GO:0043065	1.31E-02	positive regulation of apoptotic process	BP
GO:0090090	1.34E-02	negative regulation of canonical Wnt signaling pathway	BP
GO:0008380	1.43E-02	RNA splicing	BP
GO:0006283	1.47E-02	transcription-coupled nucleotide-excision repair	BP
GO:1901796	1.66E-02	regulation of signal transduction by p53 class mediator	BP
GO:0008625	2.15E-02	extrinsic apoptotic signaling pathway via death receptors	BP
GO:0043392	2.29E-02	negative regulation of DNA binding	BP
GO:0007204	2.81E-02	positive regulation of cytosolic calcium ion concentration	BP
GO:0010501	2.86E-02	RNA secondary structure unwinding	BP
GO:0000278	2.86E-02	mitotic cell cycle	BP
GO:0009611	2.94E-02	response to wounding	BP
GO:0001935	2.99E-02	endothelial cell proliferation	BP
GO:0043433	2.99E-02	negative regulation of DNA binding transcription factor	BP
GO:0030178	3.09E-02	negative regulation of Wnt signaling pathway	BP

GO:0001658	3.27E-02	branching involved in ureteric bud morphogenesis	BP
GO:0055085	3.63E-02	transmembrane transport	BP
GO:0007017	3.69E-02	microtubule-based process	BP
GO:0006869	3.75E-02	lipid transport	BP
GO:0032760	3.87E-02	positive regulation of tumor necrosis factor production	BP
GO:1901215	4.01E-02	negative regulation of neuron death	BP
GO:0042733	4.27E-02	embryonic digit morphogenesis	BP
GO:0000165	4.28E-02	MAPK cascade	BP
GO:0009411	4.86E-02	response to UV	BP
GO:0070911	4.95E-02	global genome nucleotide-excision repair	BP
GO:0051898	4.95E-02	negative regulation of protein kinase B signaling	BP
GO:0006006	5.00E-02	glucose metabolic process	BP
GO:0035098	3.63E-07	ESC/E(Z) complex	CC
GO:0005681	5.11E-03	spliceosomal complex	CC
GO:0005811	2.47E-02	lipid droplet	CC
GO:0005874	3.08E-02	microtubule	CC
GO:0043005	3.24E-02	neuron projection	CC
GO:0032580	3.52E-02	Golgi cisterna membrane	CC
GO:0001227	1.46E-05	transcriptional repressor activity	MF
GO:0000977	8.83E-04	RNA polymerase II regulatory region sequence-specific DNA	MF
GO:0000166	1.83E-03	nucleotide binding	MF
GO:0031072	9.26E-03	heat shock protein binding	MF
GO:0005525	1.67E-02	GTP binding	MF
GO:0003729	1.88E-02	mRNA binding	MF
GO:0004722	1.99E-02	protein serine/threonine phosphatase activity	MF
GO:0003684	2.21E-02	damaged DNA binding	MF
GO:0003924	2.34E-02	GTPase activity	MF
GO:0003725	2.39E-02	double-stranded RNA binding	MF
GO:0001786	3.54E-02	phosphatidylserine binding	MF

GO:0020037	3.63E-02	heme binding	MF
GO:0003677	3.80E-02	DNA binding	MF
GO:0032947	3.82E-02	protein complex scaffold activity	MF
GO:0016712	4.39E-02	oxidoreductase activity	MF
GO:0005507	4.83E-02	copper ion binding	MF

BP = biological process, CC = cell component, MF = molecular function

Supplementary Table 3. GO term enrichment for differentially expressed genes up-regulated in response to warm temperatures.

Category	*p*-value	Term	Ontology
GO:0051092	1.29E-03	positive regulation of NF-kappaB transcription factor activity	BP
GO:0006457	3.66E-03	protein folding	BP
GO:0001755	3.95E-03	neural crest cell migration	BP
GO:0051028	8.10E-03	mRNA transport	BP
GO:0042542	1.01E-02	response to hydrogen peroxide	BP
GO:0051781	1.09E-02	positive regulation of cell division	BP
GO:0043010	1.13E-02	camera-type eye development	BP
GO:0006641	1.13E-02	triglyceride metabolic process	BP
GO:1900034	1.23E-02	regulation of cellular response to heat	BP
GO:0006094	1.35E-02	gluconeogenesis	BP
GO:0034063	1.35E-02	stress granule assembly	BP
GO:0033962	1.39E-02	cytoplasmic mRNA processing body assembly	BP
GO:0009408	1.42E-02	response to heat	BP
GO:0014068	1.43E-02	positive regulation of phosphatidylinositol 3-kinase signaling	BP
GO:0032088	2.47E-02	negative regulation of NF-kappaB transcription factor activity	BP
GO:0008203	2.63E-02	cholesterol metabolic process	BP
GO:0034599	2.82E-02	cellular response to oxidative stress	BP
GO:0043044	2.91E-02	ATP-dependent chromatin remodeling	BP
GO:0098869	3.30E-02	cellular oxidant detoxification	BP
GO:0006406	3.55E-02	mRNA export from nucleus	BP
GO:0051289	3.80E-02	protein homotetramerization	BP
GO:0050821	3.83E-02	protein stabilization	BP
GO:0045454	4.57E-02	cell redox homeostasis	BP
GO:0007018	4.75E-02	microtubule-based movement	BP
GO:0006366	5.68E-02	transcription from RNA polymerase II promoter	BP

GO:0035264	6.81E-02	multicellular organism growth	BP
GO:0045740	7.16E-02	positive regulation of DNA replication	BP
GO:0050790	9.70E-02	regulation of catalytic activity	BP
GO:0006139	9.72E-02	nucleobase-containing compound metabolic process	BP
GO:0007218	9.76E-02	neuropeptide signaling pathway	BP
GO:0005788	5.58E-04	endoplasmic reticulum lumen	CC
GO:0005778	2.09E-03	peroxisomal membrane	CC
GO:0016234	4.85E-03	inclusion body	CC
GO:0042470	5.00E-03	melanosome	CC
GO:0034399	7.55E-03	nuclear periphery	CC
GO:0031527	8.03E-03	filopodium membrane	CC
GO:0005739	1.29E-02	mitochondrion	CC
GO:0031965	1.37E-02	nuclear membrane	CC
GO:0005844	1.73E-02	polysome	CC
GO:0031594	3.86E-02	neuromuscular junction	CC
GO:0030175	6.64E-02	filopodium	CC
GO:0042383	8.19E-02	sarcolemma	CC
GO:0005925	8.21E-02	focal adhesion	CC
GO:0051082	6.13E-07	unfolded protein binding	MF
GO:0031072	8.49E-04	heat shock protein binding	MF
GO:0051879	1.12E-03	Hsp90 protein binding	MF
GO:0016887	1.21E-03	ATPase activity	MF
GO:0042826	1.98E-03	histone deacetylase binding	MF
GO:0001205	6.60E-03	transcriptional activator activity, sequence-specific binding	MF
GO:0050661	6.82E-03	NADP binding	MF
GO:0017166	8.09E-03	vinculin binding	MF
GO:0003690	8.16E-03	double-stranded DNA binding	MF
GO:0003756	8.35E-03	protein disulfide isomerase activity	MF
GO:0005178	8.37E-03	integrin binding	MF

GO:0000166	2.63E-02	nucleotide binding	MF
GO:0051087	3.24E-02	chaperone binding	MF
GO:0003777	3.24E-02	microtubule motor activity	MF
GO:0016787	3.45E-02	hydrolase activity	MF
GO:0003729	3.84E-02	mRNA binding	MF
GO:0005102	6.70E-02	receptor binding	MF
GO:0042803	9.16E-02	protein homodimerization activity	MF
GO:0004725	9.31E-02	protein tyrosine phosphatase activity	MF

BP = biological process, CC = cell component, MF = molecular function

Supplementary Table 4. Genes from the DGE analysis below 0.001 significance threshold with adjusted p-values (p_{adj}), log fold changes (LFC), and the standard error in log fold change (LFC_{se}).

Gene	p_{adj}	LFC	LFC_{se}
Uncharacterized	4.76E-15	-1.25	0.14
Jumonji isoform X1	4.70E-13	-1.22	0.15
Jumonji isoform X2	1.38E-12	-1.30	0.16
Uncharacterized	9.20E-12	-1.70	0.22
DnaJ homolog subfamily A member 4	1.92E-11	4.70	0.61
High mobility group B1	1.92E-11	-1.78	0.23
Glycerol-3-phosphate mitochondrial isoform	3.57E-11	1.01	0.13
Jumonji isoform X2	5.53E-09	-1.29	0.19
Jumonji isoform X1	5.53E-09	-1.41	0.20
Uncharacterized	1.48E-07	-0.67	0.10
Heat shock cognate 71	3.20E-07	-0.84	0.13
O-case	1.23E-06	-0.67	0.11
Uncharacterized	1.37E-06	-0.93	0.15
Guanine nucleotide-binding -like 1	4.57E-06	-0.57	0.10
Heat shock HSP 90-alpha	5.72E-06	1.99	0.34
RNA-binding 5	7.68E-06	-0.55	0.10
RNA-binding 39 isoform X2	1.73E-05	-0.45	0.08
Global transcription activator SNF2L1	3.07E-05	2.07	0.38
Uncharacterized	3.14E-05	-0.51	0.09
Minor histocompatibility antigen H13 isoform	3.14E-05	-0.49	0.09
Acylamino-acid-releasing enzyme-like	3.34E-05	1.69	0.31
Catalase	4.86E-05	0.85	0.16
Uncharacterized	5.65E-05	-0.63	0.12
Cold shock domain-containing C2	6.50E-05	-0.40	0.08
F-box only 6-like	8.57E-05	0.71	0.14

Probable global transcription activator SNF2L1	9.86E-05	1.83	0.35
Uncharacterized	1.39E-04	0.87	0.17
Nuclear cap-binding subunit 2	1.39E-04	1.31	0.26
Probable aminopeptidase NPEPL1	2.13E-04	-0.46	0.09
C7orf57 like	2.42E-04	-0.69	0.14
Heterogeneous nuclear ribonucleo L-like	2.42E-04	-0.73	0.15
Serpin H1	2.64E-04	0.80	0.16
Uncharacterized	3.28E-04	-0.97	0.20
Uncharacterized	3.34E-04	-0.80	0.16
Abhydrolase domain-containing	3.34E-04	-0.88	0.18
DNA repair REV1 isoform X	3.34E-04	-0.44	0.09
Uncharacterized	3.34E-04	-0.85	0.17
UDP-glucuronic acid decarboxylase 1 isoform	4.61E-04	-0.59	0.12
DBIRD complex subunit ZNF326 isoform X1	4.61E-04	-0.44	0.09
Keratinocyte-associated transmembrane 2	4.61E-04	-0.52	0.11
Heterogeneous nuclear ribonucleo isoform	4.61E-04	1.35	0.28
RNA-binding 14 isoform X2	4.86E-04	-0.92	0.19
Uncharacterized	5.02E-04	0.66	0.14
E3 ubiquitin- ligase RNF130	5.12E-04	-0.45	0.09
Heat shock cognate 71	5.86E-04	0.75	0.16
Ropporin-1A	5.86E-04	1.52	0.32
Myelin expression factor 2 isoform X5	5.96E-04	-0.30	0.06
Cytochrome P450 3A29-like	7.57E-04	-0.66	0.14
Polypyrimidine tract-binding 1 isoform X1	7.86E-04	-0.58	0.12
Uncharacterized	9.09E-04	-0.46	0.10
Retratricopeptide repeat 39B isoform X2	9.28E-04	-0.57	0.12

BP = biological process, CC = cell component, MF = molecular function

Supplementary Table 5. Genes within the two skin resistance modules (light cyan or magenta). The gene significance (GS) and module membership (MM) along with corresponding *p*-values are identified for each gene.

Gene	Module	GS	GS *p*-value	MM	MM *p*-value
Unknown transcript	light cyan	-0.521	2.67E-03	0.776	2.92E-07
muscarinic acetylcholine receptor M3	light cyan	-0.483	5.96E-03	0.629	1.52E-04
Unknown transcript	light cyan	-0.474	7.09E-03	0.750	1.17E-06
RNA-directed DNA polymerase from mobile element jockey-like	light cyan	-0.448	1.15E-02	0.709	7.88E-06
jmjC domain-containing 8	light cyan	-0.444	1.22E-02	0.608	2.84E-04
Unknown transcript	light cyan	-0.435	1.45E-02	0.713	6.72E-06
hydroperoxide isomerase ALOXE3-like	light cyan	-0.434	1.47E-02	0.733	2.68E-06
c-binding -like	light cyan	-0.400	2.58E-02	0.820	1.66E-08
sodium potassium calcium exchanger mitochondrial	light cyan	-0.397	2.69E-02	0.708	8.42E-06
myosin light chain smooth muscle isoform X1	light cyan	-0.396	2.73E-02	0.845	2.19E-09
Unknown transcript	light cyan	-0.396	2.76E-02	0.631	1.43E-04
c-binding -like	light cyan	-0.376	3.73E-02	0.953	1.25E-16
Unknown transcript	light cyan	-0.375	3.78E-02	0.675	3.08E-05
estis-expressed sequence 2 isoform X2	light cyan	-0.364	4.40E-02	0.673	3.30E-05
Unknown transcript	light cyan	-0.363	4.49E-02	0.614	2.39E-04
protocadherin Fat 4-like	light cyan	-0.358	4.79E-02	0.807	4.30E-08
Unknown transcript	light cyan	-0.358	4.83E-02	0.806	4.53E-08
sulfotransferase 1C1-like	light cyan	-0.357	4.88E-02	0.676	2.99E-05
Unknown transcript	light cyan	-0.350	5.38E-02	0.538	1.81E-03
glutamine gamma-glutamyltransferase 6-like	light cyan	-0.343	5.89E-02	0.774	3.25E-07
Interleukin-17B	light cyan	-0.342	6.01E-02	0.682	2.37E-05
Unknown transcript	light cyan	-0.339	6.22E-02	0.752	1.05E-06
Unknown transcript	light cyan	-0.337	6.34E-02	0.741	1.88E-06
Unknown transcript	light cyan	-0.336	6.44E-02	0.676	3.03E-05

Unknown transcript	light cyan	-0.332	6.76E-02	0.598	3.76E-04
RNA-directed DNA polymerase from mobile element jockey-like	light cyan	-0.330	6.95E-02	0.679	2.70E-05
NAC-alpha domain-containing 1	light cyan	-0.328	7.16E-02	0.835	5.23E-09
selenium-binding 1	light cyan	-0.308	9.18E-02	0.654	6.62E-05
Unknown transcript	light cyan	0.296	1.06E-01	-0.643	9.67E-05
Unknown transcript	light cyan	-0.285	1.21E-01	0.680	2.59E-05
Unknown transcript	light cyan	-0.282	1.24E-01	0.660	5.40E-05
Unknown transcript	light cyan	-0.282	1.25E-01	0.879	7.53E-11
Unknown transcript	light cyan	-0.278	1.31E-01	0.859	6.45E-10
Unknown transcript	light cyan	-0.277	1.31E-01	0.757	8.26E-07
phosphatase and actin regulator 2	light cyan	0.277	1.32E-01	-0.601	3.51E-04
NAC-alpha domain-containing 1-like	light cyan	-0.275	1.35E-01	0.830	7.50E-09
LINE-1 reverse transcriptase	light cyan	-0.274	1.36E-01	0.861	5.35E-10
guanine nucleotide-binding subunit alpha-14	light cyan	-0.272	1.39E-01	0.564	9.51E-04
myosin-11 isoform X1	light cyan	-0.271	1.41E-01	0.745	1.56E-06
Unknown transcript	light cyan	-0.270	1.43E-01	0.850	1.44E-09
Unknown transcript	light cyan	-0.268	1.45E-01	0.483	5.91E-03
Unknown transcript	light cyan	-0.263	1.52E-01	0.747	1.38E-06
CASP8 and FADD-like apoptosis regulator isoform X1	light cyan	0.258	1.61E-01	-0.559	1.09E-03
zinc transporter	light cyan	0.258	1.62E-01	-0.714	6.47E-06
myosin regulatory light polypeptide 9	light cyan	-0.252	1.71E-01	0.761	6.58E-07
Unknown transcript	light cyan	0.245	1.83E-01	-0.635	1.25E-04
Unknown transcript	light cyan	-0.240	1.94E-01	0.685	2.11E-05
long-chain-fatty-acid-- ligase 4 isoform X2	light cyan	0.228	2.17E-01	-0.584	5.64E-04
Unknown transcript	light cyan	-0.227	2.19E-01	0.845	2.15E-09
type I cytoskeletal 17	light cyan	-0.226	2.21E-01	0.622	1.86E-04
immunoglobulin superfamily member 5 isoform X1	light cyan	0.222	2.29E-01	-0.709	8.03E-06
NAC-alpha domain-containing 1	light cyan	-0.222	2.29E-01	0.736	2.38E-06
Unknown transcript	light cyan	-0.220	2.34E-01	0.678	2.76E-05

peptidase inhibitor	light cyan	0.216	2.44E-01	-0.641	1.01E-04
Unknown transcript	light cyan	-0.213	2.49E-01	0.667	4.15E-05
septin-7-like isoform X4	light cyan	-0.213	2.50E-01	0.601	3.47E-04
PR domain containing	light cyan	-0.204	2.71E-01	0.641	1.01E-04
Unknown transcript	light cyan	-0.201	2.78E-01	0.700	1.19E-05
alpha-N-acetylgalactosaminide alpha-2,6-sialyltransferase 3	light cyan	-0.189	3.08E-01	0.562	1.01E-03
cysteine-rich hydrophobic domain-containing 2	light cyan	0.188	3.10E-01	-0.585	5.42E-04
Unknown transcript	light cyan	-0.172	3.54E-01	0.844	2.42E-09
Unknown transcript	light cyan	-0.168	3.67E-01	0.819	1.81E-08
Unknown transcript	light cyan	-0.165	3.74E-01	0.766	5.18E-07
cdc42 effector 3	light cyan	-0.152	4.14E-01	0.728	3.48E-06
15-hydroxyprostaglandin dehydrogenase [NAD(+)]	light cyan	-0.145	4.38E-01	0.567	8.77E-04
cytochrome P450 2F2	light cyan	0.131	4.82E-01	0.570	8.22E-04
selenium-binding 1	light cyan	-0.129	4.89E-01	0.631	1.40E-04
serine threonine- kinase pim-1	light cyan	-0.127	4.95E-01	0.684	2.18E-05
Unknown transcript	light cyan	-0.111	5.51E-01	0.687	2.01E-05
Unknown transcript	light cyan	-0.106	5.70E-01	0.809	3.56E-08
pericentrin isoform X5	light cyan	-0.102	5.86E-01	0.606	3.03E-04
Unknown transcript	light cyan	0.095	6.09E-01	-0.574	7.39E-04
Unknown transcript	light cyan	0.037	8.44E-01	0.637	1.15E-04
translocating chain-associated membrane 2	light cyan	0.012	9.50E-01	-0.520	2.70E-03
Unknown transcript	light cyan	0.011	9.54E-01	-0.237	2.00E-01
Unknown transcript	magenta	-0.514	3.11E-03	-0.557	1.14E-03
Unknown transcript	magenta	0.495	4.66E-03	0.731	2.99E-06
E3 ubiquitin- ligase TRIM63	magenta	0.487	5.42E-03	0.886	3.29E-11
RNA-directed DNA polymerase from mobile element jockey-	magenta	-0.472	7.35E-03	-0.496	4.56E-03
2-5A-dependent ribonuclease	magenta	-0.469	7.79E-03	-0.606	3.01E-04
kelch 38	magenta	0.455	1.02E-02	0.874	1.42E-10
cAMP-specific 3 ,5 -cyclic phosphodiesterase 4B isoform X1	magenta	0.444	1.24E-02	0.428	1.64E-02

tetraspanin-4 isoform X1	magenta	0.439	1.34E-02	0.569	8.30E-04
Proline-rich nuclear receptor coactivator 1	magenta	0.432	1.52E-02	0.789	1.30E-07
DNA-directed RNA polymerase III subunit RPC3	magenta	0.432	1.52E-02	0.652	6.98E-05
acyl- synthetase short-chain family member mitochondrial	magenta	-0.428	1.63E-02	-0.605	3.15E-04
Unknown transcript	magenta	-0.421	1.85E-02	-0.684	2.21E-05
tyrosine- kinase JAK2	magenta	-0.418	1.93E-02	-0.645	8.96E-05
tribbles homolog 3	magenta	0.416	1.99E-02	0.759	7.35E-07
tubulin-specific chaperone cofactor E	magenta	0.414	2.05E-02	0.683	2.27E-05
F-box only 30	magenta	0.408	2.26E-02	0.747	1.40E-06
Unknown transcript	magenta	-0.407	2.32E-02	-0.582	5.98E-04
Casein kinase II subunit alpha	magenta	0.406	2.35E-02	0.664	4.62E-05
microtubule-associated s 1A 1B light chain 3A	magenta	0.405	2.39E-02	0.850	1.45E-09
Unknown transcript	magenta	0.404	2.40E-02	0.521	2.64E-03
Unknown transcript	magenta	-0.392	2.93E-02	-0.525	2.43E-03
Unknown transcript	magenta	0.389	3.06E-02	0.707	8.89E-06
Heterogeneous nuclear ribonucleo	magenta	-0.383	3.35E-02	-0.772	3.57E-07
Unknown transcript	magenta	0.380	3.48E-02	0.611	2.62E-04
Unknown transcript	magenta	0.380	3.49E-02	0.514	3.11E-03
Unknown transcript	magenta	-0.379	3.55E-02	-0.547	1.46E-03
sodium-coupled neutral amino acid transporter 10	magenta	-0.377	3.65E-02	-0.635	1.25E-04
Unknown transcript	magenta	0.377	3.67E-02	0.605	3.13E-04
secreted frizzled-related 2-like	magenta	-0.373	3.86E-02	-0.849	1.56E-09
peripheral plasma membrane CASK isoform X8	magenta	0.371	3.97E-02	0.734	2.59E-06
ras-related Rab-10	magenta	0.370	4.08E-02	0.626	1.68E-04
Unknown transcript	magenta	0.358	4.77E-02	0.813	2.66E-08
polypyrimidine tract-binding 3 isoform X1	magenta	-0.358	4.77E-02	-0.602	3.43E-04
ruvB-like 1	magenta	0.349	5.46E-02	0.351	5.28E-02
Eukaryotic translation initiation factor 4 gamma	magenta	0.345	5.73E-02	0.847	1.97E-09
KIAA0930 homolog isoform X2	magenta	0.343	5.89E-02	0.721	4.73E-06

E3 ubiquitin- ligase RNF185	magenta	-0.340	6.11E-02	-0.483	5.96E-03
reticulophagy receptor FAM134B isoform X2	magenta	0.339	6.17E-02	0.930	3.87E-14
ATP-dependent RNA helicase DDX54 isoform X1	magenta	0.337	6.38E-02	0.701	1.12E-05
amyloid 2 isoform X2	magenta	-0.331	6.90E-02	-0.645	8.97E-05
E3 ubiquitin- ligase HERC2	magenta	0.331	6.92E-02	0.617	2.20E-04
UDP- c:betaGal beta-1,3-N-acetylglucosaminyltransferase 2	magenta	0.330	6.96E-02	0.735	2.54E-06
dipeptidyl peptidase 4	magenta	-0.329	7.04E-02	-0.320	7.90E-02
TSC22 domain family 1 isoform X1	magenta	0.327	7.25E-02	0.540	1.71E-03
RNA-directed DNA polymerase from mobile element jockey-	magenta	0.324	7.56E-02	0.753	1.01E-06
echinoderm microtubule-associated -like 4 isoform X1	magenta	-0.323	7.60E-02	-0.806	4.41E-08
coagulation factor X	magenta	0.322	7.76E-02	0.498	4.36E-03
Unknown transcript	magenta	0.320	7.90E-02	0.782	2.02E-07
tripartite motif-containing 55 isoform X1	magenta	0.316	8.33E-02	0.870	2.16E-10
E3 ubiquitin- ligase ARIH1	magenta	0.316	8.34E-02	0.781	2.14E-07
beta-Ala-His dipeptidase	magenta	-0.314	8.50E-02	-0.303	9.71E-02
disabled homolog 2 isoform X1	magenta	0.314	8.52E-02	0.798	7.81E-08
sister chromatid cohesion PDS5 homolog B isoform X1	magenta	-0.304	9.62E-02	-0.555	1.19E-03
N-acetylglucosamine-6-	magenta	0.303	9.74E-02	0.669	3.91E-05
Unknown transcript	magenta	0.303	9.77E-02	0.501	4.05E-03
PREDICTED: uncharacterized protein LOC102947292	magenta	-0.302	9.83E-02	-0.458	9.57E-03
ubiquitin-conjugating enzyme E2 R2	magenta	0.301	9.99E-02	0.645	9.00E-05
E3 ubiquitin- ligase	magenta	0.301	1.00E-01	0.705	9.52E-06
kelch-like ECH-associated 1	magenta	0.298	1.04E-01	0.778	2.64E-07
Unknown transcript	magenta	-0.295	1.08E-01	-0.762	6.46E-07
Unknown transcript	magenta	-0.293	1.10E-01	-0.681	2.47E-05
Vacuolar sorting-associated	magenta	-0.292	1.12E-01	-0.674	3.24E-05
peptidase M20 domain-containing 2	magenta	0.291	1.13E-01	0.836	4.79E-09
Unknown transcript	magenta	0.291	1.13E-01	0.711	7.23E-06
DNA polymerase alpha catalytic subunit	magenta	-0.289	1.14E-01	-0.589	4.91E-04

cap-specific mRNA (nucleoside-2 -O-)-methyltransferase 1	magenta	0.289	1.15E-01	0.453	1.04E-02
oxidative stress-responsive serine-rich 1	magenta	0.289	1.15E-01	0.774	3.29E-07
major facilitator superfamily domain-containing 4	magenta	-0.281	1.26E-01	-0.776	2.87E-07
leukocyte receptor cluster member 9	magenta	0.280	1.27E-01	0.477	6.70E-03
legumain	magenta	0.279	1.29E-01	0.840	3.39E-09
round spermatid basic 1	magenta	-0.278	1.30E-01	-0.833	5.88E-09
ETS domain-containing Elk-	magenta	0.278	1.31E-01	0.449	1.14E-02
sphingosine kinase 1	magenta	0.275	1.34E-01	0.358	4.83E-02
digestive cysteine ase 2-like	magenta	0.275	1.34E-01	0.546	1.50E-03
activin receptor type-2A isoform X2	magenta	0.274	1.36E-01	0.736	2.34E-06
collagenase 3-like	magenta	0.266	1.47E-01	0.628	1.56E-04
C-ets-2	magenta	-0.266	1.49E-01	-0.306	9.46E-02
peptide mitochondrial	magenta	0.264	1.52E-01	0.511	3.28E-03
apoptosis-associated speck containing a CARD	magenta	-0.264	1.52E-01	-0.637	1.15E-04
tetraspanin-31	magenta	0.263	1.53E-01	0.695	1.46E-05
transcription factor	magenta	0.261	1.56E-01	0.799	7.17E-08
histone-lysine N-methyltransferase EHMT1 isoform X13	magenta	-0.261	1.57E-01	-0.621	1.92E-04
WW domain-binding 1	magenta	0.258	1.60E-01	0.779	2.43E-07
uridine-cytidine kinase 2	magenta	0.256	1.65E-01	0.459	9.38E-03
dual specificity mitogen-activated kinase kinase 3	magenta	0.247	1.80E-01	0.773	3.47E-07
stromal interaction molecule 1 isoform X3	magenta	0.246	1.82E-01	0.644	9.16E-05
beta-galactosidase	magenta	0.246	1.82E-01	0.412	2.12E-02
dysbindin domain-containing 2 isoform X1	magenta	0.246	1.83E-01	0.615	2.32E-04
Unknown transcript	magenta	0.245	1.83E-01	0.572	7.83E-04
growth arrest and DNA damage-inducible GADD45 gamma	magenta	0.245	1.84E-01	0.715	6.27E-06
phosphatidylinositol-binding clathrin assembly -like isoform X4	magenta	0.242	1.89E-01	0.722	4.62E-06
peptidyl-prolyl cis-trans isomerase FKBP5	magenta	0.239	1.95E-01	0.623	1.82E-04
tetraspanin-3-like	magenta	0.239	1.96E-01	0.651	7.34E-05
Unknown transcript	magenta	-0.237	2.00E-01	-0.563	9.68E-04

cellular tumor antigen p53 isoform X1	magenta	-0.236	2.01E-01	-0.696	1.36E-05
ankyrin repeat and LEM domain-containing 2	magenta	0.235	2.02E-01	0.635	1.23E-04
zinc finger 598	magenta	0.233	2.07E-01	0.727	3.66E-06
Transcription initiation factor TFIID subunit 3	magenta	0.231	2.11E-01	0.696	1.36E-05
Inactive serine threonine- kinase VRK3	magenta	-0.231	2.11E-01	-0.593	4.41E-04
cohesin subunit SA-2 isoform X1	magenta	-0.228	2.18E-01	-0.457	9.77E-03
FAM177A1	magenta	0.227	2.19E-01	0.612	2.55E-04
phospholipid-transporting ATPase IK	magenta	-0.227	2.20E-01	-0.575	7.07E-04
calcineurin B homologous 1	magenta	0.226	2.21E-01	0.742	1.76E-06
Unknown transcript	magenta	-0.222	2.31E-01	-0.570	8.24E-04
sorting nexin-32 isoform X1	magenta	-0.221	2.33E-01	-0.453	1.06E-02
type 2 lactosamine alpha-2,3-sialyltransferase isoform X2	magenta	-0.220	2.35E-01	-0.628	1.54E-04
sestrin-1 isoform X2	magenta	0.218	2.38E-01	0.683	2.26E-05
heterochromatin 1-binding 3 isoform X2	magenta	-0.218	2.40E-01	-0.810	3.47E-08
AP kinase-interacting serine threonine- kinase 2	magenta	0.211	2.55E-01	0.584	5.63E-04
WNT1-inducible-signaling pathway 1	magenta	-0.210	2.57E-01	-0.513	3.20E-03
Unknown transcript	magenta	-0.210	2.58E-01	-0.422	1.80E-02
lifeguard 1	magenta	0.207	2.65E-01	0.712	7.09E-06
Unknown transcript	magenta	-0.206	2.67E-01	-0.686	2.07E-05
Unknown transcript	magenta	0.204	2.70E-01	0.809	3.66E-08
ubiquitin-conjugating enzyme E2 Z	magenta	0.203	2.74E-01	0.465	8.36E-03
nuclear prelamin A recognition factor	magenta	0.203	2.74E-01	0.695	1.45E-05
otopetrin-2	magenta	-0.202	2.76E-01	-0.806	4.38E-08
Unknown transcript	magenta	-0.201	2.79E-01	-0.732	2.81E-06
Unknown transcript	magenta	0.196	2.90E-01	0.724	4.11E-06
Vascular non-inflammatory molecule	magenta	0.196	2.92E-01	0.369	4.10E-02
apoptosis facilitator Bcl-2 14	magenta	-0.193	2.97E-01	-0.310	8.92E-02
EF-hand calcium-binding domain-containing 14-like	magenta	-0.193	2.98E-01	-0.785	1.76E-07
transport Sec24D	magenta	-0.192	3.00E-01	-0.396	2.73E-02

Unknown transcript	magenta	0.190	3.05E-01	0.500	4.21E-03
PWWP domain-containing 2B	magenta	-0.190	3.07E-01	-0.484	5.83E-03
Unknown transcript	magenta	0.189	3.09E-01	0.790	1.27E-07
26S proteasome non-ATPase regulatory subunit 1	magenta	0.188	3.11E-01	0.679	2.70E-05
rap guanine nucleotide exchange factor 2 isoform X2	magenta	0.188	3.12E-01	0.465	8.39E-03
arrestin domain-containing	magenta	0.183	3.24E-01	0.612	2.55E-04
cip1-interacting zinc finger	magenta	0.180	3.31E-01	0.792	1.11E-07
MAP kinase-interacting serine threonine- kinase 2	magenta	0.179	3.35E-01	0.684	2.19E-05
Unknown transcript	magenta	-0.178	3.39E-01	-0.697	1.31E-05
otopetrin-2	magenta	-0.178	3.39E-01	-0.799	7.13E-08
large neutral amino acids transporter small subunit 4 isoform X1	magenta	0.176	3.45E-01	0.708	8.40E-06
Unknown transcript	magenta	0.175	3.47E-01	0.662	5.00E-05
SSUH2 homolog isoform X2	magenta	-0.174	3.51E-01	-0.364	4.39E-02
sorbitol dehydrogenase	magenta	0.170	3.62E-01	0.405	2.36E-02
epithelial chloride channel -like	magenta	-0.170	3.62E-01	-0.541	1.68E-03
tripartite motif-containing 7-like	magenta	0.166	3.73E-01	0.761	6.71E-07
Unknown transcript	magenta	0.164	3.79E-01	0.485	5.71E-03
Unknown transcript	magenta	-0.164	3.79E-01	-0.777	2.68E-07
Unknown transcript	magenta	-0.162	3.84E-01	0.318	8.11E-02
phospholipid scramblase 3 isoform X2	magenta	0.160	3.90E-01	0.330	7.02E-02
type I cytoskeletal 12-like isoform X2	magenta	-0.160	3.91E-01	-0.615	2.33E-04
olyhomeotic 3	magenta	0.159	3.91E-01	0.625	1.69E-04
c-binding -like	magenta	-0.158	3.96E-01	-0.333	6.70E-02
Semaphorin-	magenta	-0.158	3.97E-01	-0.603	3.28E-04
sestrin-3-like isoform X1	magenta	0.156	4.01E-01	0.737	2.28E-06
charged multivesicular body 4b	magenta	0.155	4.05E-01	0.525	2.43E-03
exocyst complex component 7 isoform X3	magenta	0.154	4.07E-01	0.629	1.51E-04
Unknown transcript	magenta	-0.153	4.12E-01	-0.452	1.07E-02
carnitine O-palmitoyltransferase liver isoform	magenta	-0.149	4.24E-01	-0.705	9.41E-06

clusterin	magenta	0.146	4.33E-01	0.790	1.28E-07
semaphorin-3E	magenta	-0.143	4.42E-01	-0.693	1.53E-05
decapping and exoribonuclease	magenta	-0.140	4.53E-01	-0.654	6.50E-05
NPAT isoform X2	magenta	-0.138	4.59E-01	-0.681	2.46E-05
adseverin isoform X1	magenta	-0.135	4.70E-01	-0.497	4.49E-03
transcobalamin-2	magenta	0.134	4.73E-01	0.630	1.46E-04
MOB kinase activator 2 isoform X1	magenta	0.132	4.80E-01	0.614	2.37E-04
cytosolic non-specific dipeptidase isoform X1	magenta	0.131	4.84E-01	0.479	6.39E-03
rap guanine nucleotide exchange factor 1 isoform X7	magenta	0.119	5.25E-01	0.523	2.54E-03
endonuclease 8-like 1	magenta	-0.118	5.27E-01	-0.309	9.08E-02
pro-cathepsin H	magenta	0.116	5.36E-01	-0.354	5.05E-02
Unknown transcript	magenta	0.112	5.49E-01	0.490	5.11E-03
Unknown transcript	magenta	-0.111	5.51E-01	-0.428	1.64E-02
Unknown transcript	magenta	-0.111	5.53E-01	0.334	6.59E-02
NXPE family member 1 isoform X1	magenta	-0.111	5.54E-01	-0.375	3.78E-02
sulfate transporter-like	magenta	-0.110	5.56E-01	-0.385	3.23E-02
RNA-directed DNA polymerase from mobile element jockey-	magenta	0.107	5.67E-01	0.623	1.81E-04
probable ATP-dependent RNA helicase DDX27	magenta	0.106	5.70E-01	0.729	3.29E-06
solute carrier family 28 member 3	magenta	-0.095	6.11E-01	-0.514	3.12E-03
Unknown transcript	magenta	-0.094	6.17E-01	-0.580	6.22E-04
tapasin	magenta	-0.093	6.17E-01	-0.439	1.35E-02
Unknown transcript	magenta	-0.089	6.33E-01	0.386	3.19E-02
dual specificity mitogen-activated kinase kinase 1	magenta	-0.089	6.35E-01	-0.633	1.32E-04
signal recognition particle subunit SRP68	magenta	0.089	6.35E-01	0.602	3.35E-04
large neutral amino acids transporter small subunit 4 isoform X1	magenta	0.088	6.38E-01	0.625	1.69E-04
SH3 domain-binding	magenta	-0.080	6.70E-01	0.329	7.05E-02
aldose reductase-like	magenta	-0.077	6.82E-01	-0.655	6.31E-05
Unknown transcript	magenta	0.076	6.83E-01	0.646	8.69E-05
ferritin light oocyte isoform-like	magenta	0.076	6.83E-01	0.543	1.59E-03

Unknown transcript	magenta	-0.071	7.03E-01	-0.706	9.17E-06
regulatory factor X-associated	magenta	-0.070	7.07E-01	-0.402	2.52E-02
G pathway suppressor 2	magenta	0.065	7.26E-01	0.694	1.48E-05
Zinc finger	magenta	-0.065	7.30E-01	-0.523	2.55E-03
Unknown transcript	magenta	-0.054	7.71E-01	-0.667	4.11E-05
Unknown transcript	magenta	-0.051	7.86E-01	-0.313	8.66E-02
Unknown transcript	magenta	-0.050	7.89E-01	-0.450	1.11E-02
NF-kappa-B essential modulator	magenta	0.039	8.33E-01	0.506	3.71E-03
C6orf62 like	magenta	0.037	8.41E-01	0.518	2.82E-03
Unknown transcript	magenta	0.026	8.91E-01	-0.505	3.77E-03
Unknown transcript	magenta	0.023	9.02E-01	0.540	1.73E-03
ubiquitin-like modifier-activating enzyme 1	magenta	-0.023	9.04E-01	-0.466	8.17E-03
Unknown transcript	magenta	0.019	9.19E-01	0.583	5.79E-04
ferric-chelate reductase 1	magenta	-0.018	9.23E-01	-0.573	7.57E-04
reverse transcriptase	magenta	-0.018	9.23E-01	-0.359	4.73E-02
lanosterol 14-alpha demethylase	magenta	0.014	9.41E-01	-0.431	1.56E-02
THAP domain-containing 4 isoform X2	magenta	0.011	9.53E-01	0.426	1.69E-02
Unknown transcript	magenta	-0.005	9.78E-01	-0.502	3.97E-03
Unknown transcript	magenta	-0.003	9.86E-01	-0.464	8.53E-03
Unknown transcript	magenta	-0.003	9.86E-01	-0.600	3.63E-04
nucleotide exchange factor SIL1	magenta	0.003	9.89E-01	0.430	1.57E-02
RNA pseudouridylate synthase domain-containing 1	magenta	0.000	9.98E-01	0.448	1.14E-02

Supplementary Table 6. Module expression is associated with the change in r_i, but not temperature and humidity. The following analysis was conducted using an ANCOVA on the module that positively correlated to the change in r_i.

**predictor**	**SS**	***df***	***F***	***p***	$\boldsymbol{\omega}^2$
**Module expression**	**4.82**	**1**	**4.45**	**0.04**	**0.10**
Temperature	0.61	1	0.57	0.45	<0.01
Humidity	0.63	1	0.59	0.45	<0.01
Module expression x temperature	0.81	1	0.75	0.39	<0.01
Module expression x humidity	0.04	1	0.03	0.85	<0.01
Temperature x humidity	0.00	1	0.00	0.99	<0.01
Module expression x temperature x humidity	0.13	1	0.12	0.73	<0.01
residuals	24.9	23			

SS = sum of squares, $\boldsymbol{\omega}^2$ = effect size

Supplementary Table 7. Module expression is associated with the change in r_i, but not temperature and humidity. The following analysis was conducted using an ANCOVA on the module that negatively correlated to the change in r_i.

**predictor**	**SS**	***df***	***F***	***p***	$\boldsymbol{\omega}^2$
**Module expression**	**5.62**	**1**	**5.51**	**0.02**	**0.11**
Temperature	1.77	1	1.73	0.20	0.02
Humidity	0.08	1	0.08	0.77	<0.01
Module expression x temperature	0.22	1	0.21	0.64	<0.01
Module expression x humidity	0.00	1	0.01	0.93	<0.01
Temperature x humidity	0.05	1	0.05	0.82	<0.01
Module expression x temperature x humidity	1.49	1	1.45	0.24	0.01
residuals	23.5	23			

SS = sum of squares, $\boldsymbol{\omega}^2$ = effect size

Supplementary Table 8. GO term enrichment for the skin resistance modules revealed functionally important processes such as vascular development and skin development.

Category	*p*-value	Term	Ontology
GO:0016525	3.37E-03	negative regulation of angiogenesis	BP
GO:0006954	9.73E-03	inflammatory response	BP
GO:0007166	1.31E-02	cell surface receptor signaling pathway	BP
GO:0007368	2.00E-02	determination of left/right symmetry	BP
GO:0042981	2.25E-02	regulation of apoptotic process	BP
GO:0043473	2.56E-02	pigmentation	BP
GO:0001676	2.59E-02	long-chain fatty acid metabolic process	BP
GO:0010862	2.81E-02	positive regulation of pathway-restricted SMAD protein phosphorylation	BP
GO:1903146	2.81E-02	regulation of autophagy of mitochondrion	BP
GO:0032486	2.83E-02	Rap protein signal transduction	BP
GO:2000249	2.94E-02	regulation of actin cytoskeleton reorganization	BP
GO:0098779	2.96E-02	positive regulation of mitophagy in response to mitochondrial depolarization	BP
GO:0015031	3.25E-02	protein transport	BP
GO:0009966	3.30E-02	regulation of signal transduction	BP
GO:0001569	3.31E-02	branching involved in blood vessel morphogenesis	BP
GO:0071320	3.31E-02	cellular response to cAMP	BP
GO:0071526	3.36E-02	semaphorin-plexin signaling pathway	BP
GO:0030335	3.80E-02	positive regulation of cell migration	BP
GO:0000785	4.37E-03	chromatin	CC
GO:0031463	1.15E-02	Cul3-RING ubiquitin ligase complex	CC
GO:0031519	1.86E-02	PcG protein complex	CC
GO:0000145	2.68E-02	exocyst	CC
GO:0030136	3.34E-02	clathrin-coated vesicle	CC
GO:0004842	7.52E-03	ubiquitin-protein transferase activity	MF
GO:0008236	7.76E-03	serine-type peptidase activity	MF

GO:0050699	9.72E-03	WW domain binding	MF
GO:0030215	1.60E-02	semaphorin receptor binding	MF
GO:0004702	1.66E-02	signal transducer with serine/threonine kinase activity	MF
GO:0004860	2.74E-02	protein kinase inhibitor activity	MF
GO:0005545	2.99E-02	1-phosphatidylinositol binding	MF

BP = biological process, CC = cell component, MF = molecular function

Supplementary Table 9. Hub genes from modules significantly associated with the change in r_i. Genes in bold are associated with an aspect of vasoconstriction or lipid barrier formation.

Gene	GS	pGS	MM	pMM
**muscarinic acetylcholine receptor M3**	**-4.83E-01**	**5.96E-03**	**6.29E-01**	**1.52E-04**
RNA-directed DNA polymerase from mobile element jockey-like	-4.48E-01	1.15E-02	7.09E-01	7.88E-06
jmjC domain-containing 8	-4.44E-01	1.22E-02	6.08E-01	2.84E-04
**hydroperoxide isomerase ALOXE3-like**	**-4.34E-01**	**1.47E-02**	**7.33E-01**	**2.68E-06**
c-binding -like	-4.00E-01	2.58E-02	8.20E-01	1.66E-08
sodium potassium calcium exchanger mitochondrial	-3.97E-01	2.69E-02	7.08E-01	8.42E-06
myosin light chain smooth muscle isoform X1	-3.96E-01	2.73E-02	8.45E-01	2.19E-09
c-binding -like	-3.76E-01	3.73E-02	9.53E-01	1.25E-16
estis-expressed sequence 2 isoform X2	-3.64E-01	4.40E-02	6.73E-01	3.30E-05
protocadherin Fat 4-like	-3.58E-01	4.79E-02	8.07E-01	4.30E-08
sulfotransferase 1C1-like	-3.57E-01	4.88E-02	6.76E-01	2.99E-05
RNA-directed DNA polymerase from mobile element jockey-	-4.72E-01	7.35E-03	-4.96E-01	4.56E-03
2-5A-dependent ribonuclease	-4.69E-01	7.79E-03	-6.06E-01	3.01E-04
acyl-synthetase short-chain family member mitochondrial	-4.28E-01	1.63E-02	-6.05E-01	3.15E-04
tyrosine- kinase JAK2	-4.18E-01	1.93E-02	-6.45E-01	8.96E-05
heterogeneous nuclear ribonucleo	-3.83E-01	3.35E-02	-7.72E-01	3.57E-07
sodium-coupled neutral amino acid transporter 10	-3.77E-01	3.65E-02	-6.35E-01	1.25E-04
secreted frizzled-related 2-like	-3.73E-01	3.86E-02	-8.49E-01	1.56E-09
polypyrimidine tract-binding 3 isoform X1	-3.58E-01	4.77E-02	-6.02E-01	3.43E-04
ras-related Rab-10	3.70E-01	4.08E-02	6.26E-01	1.68E-04
peripheral plasma membrane CASK isoform X8	3.71E-01	3.97E-02	7.34E-01	2.59E-06
microtubule-associated s 1A 1B light chain 3A	4.05E-01	2.39E-02	8.50E-01	1.45E-09
casein kinase II subunit alpha	4.06E-01	2.35E-02	6.64E-01	4.62E-05
F-box only 30	4.08E-01	2.26E-02	7.47E-01	1.40E-06

tubulin-specific chaperone cofactor E	4.14E-01	2.05E-02	6.83E-01	2.27E-05
tribbles homolog 3	4.16E-01	1.99E-02	7.59E-01	7.35E-07
DNA-directed RNA polymerase III subunit RPC3	4.32E-01	1.52E-02	6.52E-01	6.98E-05
proline-rich nuclear receptor coactivator 1	4.32E-01	1.52E-02	7.89E-01	1.30E-07
tetraspanin-4 isoform X1	4.39E-01	1.34E-02	5.69E-01	8.30E-04
**cAMP-specific 3,5-cyclic phosphodiesterase 4B isoform X1**	**4.44E-01**	**1.24E-02**	**4.28E-01**	**1.64E-02**
kelch 38	4.55E-01	1.02E-02	8.74E-01	1.42E-10
**E3 ubiquitin- ligase TRIM63**	**4.87E-01**	**5.42E-03**	**8.86E-01**	**3.29E-11**

Bolded genes have GO terms associated with water loss

Supplementary Table 10. GSEA analysis for up- and downregulated pathways in response to temperature. For each pathway, we identified the GO term, description, ontology, normalization enrichment score (NES), false discovery rate (FDR) q-value, and familywise error rate (FWER) *p*-value.

**GO term**	**Description**	**Ontology**	**NES**	**FDR *q*-value**	**FWER *p***
Upregulated					
GO:0007519	skeletal muscle tissue development	BP	1.956	0.021	0.043
GO:0006936	muscle contraction	BP	1.975	0.031	0.034
GO:0030018	z-disc	CC	1.899	0.039	0.121
GO:0051015	actin filament binding	MF	1.865	0.047	0.188
GO:0003774	motor activity	MF	1.814	0.066	0.365
GO:0042383	sarcolemma	CC	1.817	0.077	0.352
Downregulated					
GO:0048538	thymus development	BP	-2.323	0.000	0.000
GO:0001889	liver development	BP	-2.142	0.005	0.008
GO:0000977	RNA Polymerase II	MF	-2.036	0.015	0.040

Supplementary Table 11. GSEA analysis for up- and downregulated pathways in response to humidity. For each pathway, we identified the GO term, description, ontology, normalization enrichment score (NES), false discovery rate (FDR) q-value, and familywise error rate (FWER) *p*-value.

**GO term**	**Description**	**Ontology**	**NES**	**FDR *q*-value**	**FWER *p***
Upregulated					
GO:0006936	Muscle contraction	BP	2.000	0.032	0.032
GO:0030018	Z-disc	CC	1.923	0.050	0.095
Downregulated					
GO:0035023	Rho protein signal transduction	BP	-2.003	0.016	0.030
GO:0031234	Extrinsic component of membrane	CC	-2.038	0.018	0.017
GO:0005604	Basement membrane	CC	-1.954	0.025	0.069
GO:0005096	GTPase activator activity	MF	-1.910	0.035	0.126
GO:0044767	Developmental process	BP	-1.866	0.054	0.229

Supplementary Table 12. Overlap in genes between GSEA and WGCNA. The table identifies differentially expressed genes that were involved in GSEA enriched gene sets. The table contains the gene name (Gene), the GO term associated with the enrichment gene set (GO term), the description of the GO term, the treatment associated with the GSEA analysis, and the regulation of the gene set from the GSEA analysis.

Gene	GO term	Description	Treatment	Response
C-binding -like	GO:0044767	Developmental process	humidity	down
Tyrosine- kinase JAK2	GO:0031234	Extrinsic component of membrane	humidity	down
Phosphodiesterase 4B	GO:0030018	Z-disc	temperature	up
Myosin polypeptide	GO:0030018	Z-disc	temperature	up
Adseverin isoform	GO:0051015	Actin filament binding	temperature	up
Myosin-11 isoform	GO:0051015	Actin filament binding	temperature	up
Mitogen-activated kinase	GO:0048538	Thymus development	temperature	down
Rap guanine exchange factor	GO:0005096	GTPase activator activity	humidity	down

Supplementary Table 13. Overlap in genes between GSEA and DGE. The table contains differentially expressed genes that were also found in the GSEA enriched gene sets. The table identifies the gene name (Gene), the GO term associated with the enrichment gene set (GO term), the description of the GO term, the treatment associated with the GSEA analysis, and the regulation of the gene set from the GSEA analysis.

Gene	GO term	Description	Treatment	Response
Unconventional myosin	GO:0003774	Motor activity	temperature	up
Cyclic AMP transcription factor	GO:0000977	RNA polymerase II regulatory region	temperature	down
Mitogen-activated kinase 14	GO:0007519	Skeletal muscle tissue development	temperature	up
Dual specificity mitogen	GO:0048538	Thymus development	temperature	down
Jumonji isoform X2	GO:0001889	Liver development	temperature	down
Jumonji isoform X2	GO:0048538	Thymus development	temperature	down
Jumonji isoform X2	GO:0000977	RNA polymerase II regulatory region	temperature	down
Rab GTPase-activating	GO:0005096	GTPase activator activity	humidity	down
Utrophin isoform X2	GO:0042383	Sarcolemma	temperature	up
Rab GTPase-binding effector	GO:0005096	GTPase activator activity	humidity	down
B-cell lymphoma 6	GO:0000977	RNA polymerase II regulatory region	temperature	down
Unc-45 homolog B	GO:0007519	Skeletal muscle tissue development	temperature	up
Unc-45 homolog B	GO:0030018	Z-disc	temperature	up
Caveolin 1	GO:0007519	Skeletal muscle tissue development	temperature	up
Utrophin isoform X1	GO:0042383	Sarcolemma	temperature	up
Core histone macro-	GO:0000977	RNA polymerase II regulatory region	temperature	down
Jumonji isoform X1	GO:0048538	Thymus development	temperature	down
Jumonji isoform X1	GO:0000977	RNA polymerase II regulatory region	temperature	down
Jumonji isoform X1	GO:0001889	Liver development	temperature	down
Unconventional myosin	GO:0003774	Motor activity	temperature	up

Supplementary Table 14. Overlap in genes between WGCNA and DGE. The table identifies differentially expressed genes that were identified in the WGCNA. The table contains the gene significance (GS) as well as the module membership (MM), which were determined in the WGCNA.

Gene	GS	GS p-value	MM	MM p-value
Cysteine-rich hydrophobic domain	0.19	0.31	-0.58	5.4×10^{-4}
NAC-alpha domain-containing	-0.22	0.23	0.73	2.4×10^{-6}
Amyloid 2 isoform 2	-0.33	0.07	-0.64	8.9×10^{-5}
Long-chain-fatty-acid ligase 4	0.22	0.22	-0.58	5.6×10^{-4}

GS = gene significance, MM = module membership
