# Supplementary material for: Thermal cues drive plasticity of desiccation resistance in montane salamanders with implications for climate change
Source: Nat Commun. 2019 Sep 9;10:4091. doi: 10.1038/s41467-019-11990-4 (PMC6733842; doi:10.1038/s41467-019-11990-4)
Supplement: Supplementary file 3 — Reporting Summary [file 41467_2019_11990_MOESM3_ESM.pdf]

nature research

Corresponding author(s): Eric Riddell

Last updated by author(s): Jul 25, 2019

Reporting Summary

Nature Research wishes to improve the reproducibility of the work that we publish. This form provides structure for consistency and transparency in reporting. For further information on Nature Research policies, see Authors & Referees and the Editorial Policy Checklist.

Statistics

For all statistical analyses, confirm that the following items are present in the figure legend, table legend, main text, or Methods section.

n/a	Confirmed	
☐	☒	The exact sample size (n) for each experimental group/condition, given as a discrete number and unit of measurement
☐	☒	A statement on whether measurements were taken from distinct samples or whether the same sample was measured repeatedly
☐	☒	The statistical test(s) used AND whether they are one- or two-sided<br>*Only common tests should be described solely by name; describe more complex techniques in the Methods section.*
☐	☒	A description of all covariates tested
☐	☒	A description of any assumptions or corrections, such as tests of normality and adjustment for multiple comparisons
☐	☒	A full description of the statistical parameters including central tendency (e.g. means) or other basic estimates (e.g. regression coefficient) AND variation (e.g. standard deviation) or associated estimates of uncertainty (e.g. confidence intervals)
☐	☒	For null hypothesis testing, the test statistic (e.g. F, t, r) with confidence intervals, effect sizes, degrees of freedom and P value noted<br>*Give P values as exact values whenever suitable.*
☒	☐	For Bayesian analysis, information on the choice of priors and Markov chain Monte Carlo settings
☒	☐	For hierarchical and complex designs, identification of the appropriate level for tests and full reporting of outcomes
☐	☒	Estimates of effect sizes (e.g. Cohen's d, Pearson's r), indicating how they were calculated

Our web collection on statistics for biologists contains articles on many of the points above.

Software and code

Policy information about availability of computer code

Data collection	For the cues analysis, we used the recently published TerraClim dataset (http://www.climatologylab.org/products.html) of global VPDs and temperature data. These files are currently available for download on their public repository linked above.
Data analysis	We used R (v. 3.4.2.) for our statistical analyses on the physiological and environmental data. We also relied heavily upon DeSeq2, WGCNA, and GOseq libraries for our analyses on gene expression, which were carried out in the same version of R. In preparation for de novo transcriptome assembly, we trimmed Illumina-specific sequences and adapters using Trimmomatic (v. 0.36). We evaluated error probabilities of each read with ConDeTri (v. 2.3) and low-quality bases or sections of multiple bases were removed using default parameters. We assembled our de novo transcriptome using Trinity (version 2.4.0) using standard protocols for strand specific analysis and normalization. We used TransDecoder (version 3.0.1) to reduce the transcriptome to coding regions from all transcript sequences. RSEM (version 1.3.0) estimated gene and gene isoform expression levels by aligning individual samples back to the transcriptome using Bowtie (version 1.2.1.1). For the cues analyses, we used the recently published TerraClim dataset of global VPDs and temperature data. Then we used custom script in Python (v. 3.6) to analyze correlations between climatic variables. Each collection point was generated using a random point generator on QGIS (v.2.1).

For manuscripts utilizing custom algorithms or software that are central to the research but not yet described in published literature, software must be made available to editors/reviewers. We strongly encourage code deposition in a community repository (e.g. GitHub). See the Nature Research guidelines for submitting code & software for further information.

Data

Policy information about availability of data

All manuscripts must include a data availability statement. This statement should provide the following information, where applicable:

- Accession codes, unique identifiers, or web links for publicly available datasets
- A list of figures that have associated raw data
- A description of any restrictions on data availability

We provided the data required to interpret, replicate, and build upon our findings. We provided the raw data on the Open Science Framework repository (https://

osf.io/rnsmk/). The data are referenced using the following identifier: DOI 10.17605/OSF.IO/RNSMK. Figure 2 requires the available data from http://www.climatologylab.org/products.html. RNA-seq data is currently available for download on Genbank (BioProject:PRJNA509078 [https://www.ncbi.nlm.nih.gov/bioproject/PRJNA509078]).

Field-specific reporting

Please select the one below that is the best fit for your research. If you are not sure, read the appropriate sections before making your selection.

☐ Life sciences ☐ Behavioural & social sciences ☒ Ecological, evolutionary & environmental sciences

For a reference copy of the document with all sections, see nature.com/documents/nr-reporting-summary-flat.pdf

Ecological, evolutionary & environmental sciences study design

All studies must disclose on these points even when the disclosure is negative.

Study description	We conducted physiological experiences on 132 salamanders, separated across 4 treatments (warm,wet; warm,dry; cool,wet; cool, dry) in a full factorial experiment. 12 individuals were used as a baseline control and the other 120 individuals were distributed randomly and equally across the treatments. We analyzed the effect of mass and temperature treatment and their interaction for the physiological data. We also analyzed the impact of initial physiological status using standard null-hypothesis testing and analysis of covariance statistical approaches. We also analyzed environmental cues (temperature and vapor pressure deficit) using one-way type-II analysis of covariance that we recorded with iButtons distributed randomly across the study site. We analyzed gene expression from 8 randomly selected individuals from each of the treatments for subsequent analyses. We then used various techniques to identify differential expression and functionally important gene networks. Finally, we assessed global correlations between temperature and vapor pressure deficits to demonstrate the relevance of thermal cues for organisms across the planet.
Research sample	We collected the gray-cheeked salamander (Plethodon metcalfi) from the Nantahala National Forest in North Carolina. We studied this species because of the extensive physiological research that has been conducted on this species by Riddell et al. We attempted to control for age of the salamanders by collecting individuals that ranged from 2.5 to 4 g. The range was specifically designed to avoid juveniles.
Sampling strategy	The individuals were collected from randomly generated coordinates off of a gravel road that rose from 700m to 1700m at the field site. The purpose of collecting at random locations was to ensure that we collected a random representation of the population, defined as individuals located along the elevational gradient of our field site. We ensured that each treatment had at least thirty individuals. We selected thirty individuals based on the limitations of our ability to expose individuals to their treatments and previously collected data that suggested 30 individuals was sufficient to detect changes in plasticity of skin resistance to water loss. Sexes were not known, but previous studies have not found significant differences in sex in terms of resistance to water loss. We sampled evenly across elevation (low, mid, and high) to evaluate potential local adaptation that had been reported in earlier work. Each individual was randomly assigned to their treatment with respect to the elevation from which they were captured.
Data collection	Riddell collected the salamanders from the field site. Riddell measured the physiological traits using a flow-through respirometry system in the lab at Clemson University. Riddell extracted, purified, quantification, and prepared the RNA libraries for sequencing at Cornell University under the guidance of Zamudio. Riddell, Roback, and Wells constructed the transcriptome. Riddell analyzed the physiological experiments, environmental data, and gene expression data. Riddell and Sears designed the experiments. Riddell wrote the manuscript. Sears provided edits to the manuscript.
Timing and spatial scale	We collected the individuals from the field over a five day period. The collection began May 17th 2016 and ended May 21st 2016. We elected to use this time scale of collection to ensure that each individual was in the lab for the exact same amount of time when undergoing exposure to the laboratory conditions. The individuals were collected over a 10km x 10km area at the field site. We elected to use this spatial extent to capture variation in elevation that had been investigated previously, specifically on geographic variation in water loss physiology.
Data exclusions	All data were included in the final analyses of the physiological and environmental data. For the gene expression analysis, we removed one outlier sample based upon criteria in the WGCNA analysis.
Reproducibility	We found our environmental analyses to reproducible across years in the experiment. This was the second time that we had conducted this experiment, and although the first experiment was not published, we found very similar results. We also found reproducible results between different analyses using differential gene expression and weight-gene co-expression analyses. These two independent analyses produced the same conclusions.
Randomization	We used randomization throughout our study using the random library from Python. Individuals were randomly assigned to a treatment based upon their elevation from which they were captured and their mass. This ensured that each treatment consisted of salamanders of the same size and equally represented across elevation. We also randomly assigned individuals within a treatment to a random shelf every day of the experiment. Their location in the incubator was adjusted randomly in the shelf as well each time that we adjusted the shelf. We also assigned a treatment to a random incubator switching the treatment with the incubator). Therefore, individuals experienced every incubator, shelf, and position on a shelf by the end of the experiment. This ensured that these factors did not confound our experiments. Finally, we randomly assigned individual samples to each lane to be sequenced based upon treatment and tissue type. This ensured that any differences were due to the experiment and not the lane on which the sample was sequenced.

Blinding	Researchers were blind to the treatment that each individual would be assigned, as this was done in random simulations. Due to the high number of individuals being removed from their treatments on a daily basis, Riddell could not be blind to the treatment or ID of the animal. The extent of randomization would have made human error very likely.

Did the study involve field work? ☒ Yes ☐ No

Field work, collection and transport

Field conditions	We report extensively on the temperature and humidity in the manuscript. Please see the manuscript for those highly specific details. Otherwise, the field conditions can be described as a temperate rain forest. It's generally cool (~20C) and it often rains every day at the field site in the summer.
Location	The salamanders were collected in the Nantahala National Forest (35˚ 20’ N, 83˚ 4’ W).
Access and import/export	We approved our experiments with the Institute for Animal Care and Use Committee at Clemson University (#2014-024), and collections were approved by the North Carolina Wildlife Commission (#16-SC00746) and United States Fish and Wildlife Service (#MA90761B-0). The North Carolina Wildlife Commission gave us access to the field site.
Disturbance	Sampling caused very minimal disturbance by turning over logs, occasionally. We minimized this disturbance by sampling at night when salamanders are commonly found walking around on the forest floor. We always made sure that logs were placed gently back in their original location and configuration.

Reporting for specific materials, systems and methods

We require information from authors about some types of materials, experimental systems and methods used in many studies. Here, indicate whether each material, system or method listed is relevant to your study. If you are not sure if a list item applies to your research, read the appropriate section before selecting a response.

Materials & experimental systems

n/a	Involved in the study	
☐	☐	Antibodies
☐	☐	Eukaryotic cell lines
☐	☐	Palaeontology
☐	☒	Animals and other organisms
☐	☐	Human research participants
☐	☐	Clinical data

Methods

n/a	Involved in the study	
☐	☐	ChIP-seq
☐	☐	Flow cytometry
☐	☐	MRI-based neuroimaging

Antibodies

Antibodies used	We did not use antibodies.
Validation	We did not use antibodies.

Eukaryotic cell lines

Policy information about cell lines

Cell line source(s)	We did not use eukaryotic cell lines.
Authentication	We did not use eukaryotic cell lines.
Mycoplasma contamination	We did not use eukaryotic cell lines.
Commonly misidentified lines (See ICLAC register)	We did not use eukaryotic cell lines.

Palaeontology

Specimen provenance	Paleontology was not involved in our study.
Specimen deposition	Paleontology was not involved in our study.
Dating methods	Paleontology was not involved in our study.

☐ Tick this box to confirm that the raw and calibrated dates are available in the paper or in Supplementary Information.

Animals and other organisms

Policy information about studies involving animals; ARRIVE guidelines recommended for reporting animal research

Laboratory animals	Plethodon metcalfi; sex was unknown; exact age was unknown but all salamanders were adults.
Wild animals	Plethodon metcalfi was captured by hand. Individuals were placed in Ziploc bags with moist leaf litter and transported back to the laboratory in Clemson, SC on the same night of capture. After the study, salamanders were humanely euthanized after the end of the experiment to extract total RNA for genomic purposes.
Field-collected samples	Salamanders were housed in individual plastic containers (17 cm x 17 cm x 12 cm) with moist paper towels for rehydration and fed crickets (Acheta domesticus) ad libitum throughout the month-long acclimation period. All salamanders were maintained in a Percival incubator (Percival, Inc.; Model #I-36VL) a cool, cycling thermal regime (10-15C) for one month to acclimate to laboratory conditions. The cycling thermal regime was designed to mimic conditions that salamanders experience in the early spring.
Ethics oversight	The Institute for Animal Care and Use Committee at Clemson University (#2014-024) approved our study and ensured that the salamanders were healthy. We also had protocols to ensure salamanders were healthy by visual inspection during the experiment. We also ensured that salamanders maintained a healthy mass during the experiment.

Note that full information on the approval of the study protocol must also be provided in the manuscript.

Human research participants

Policy information about studies involving human research participants

Population characteristics	Humans were not used.
Recruitment	Humans were not used.
Ethics oversight	Humans were not used.

Note that full information on the approval of the study protocol must also be provided in the manuscript.

Clinical data

Policy information about clinical studies

All manuscripts should comply with the ICMJE guidelines for publication of clinical research and a completed CONSORT checklist must be included with all submissions.

Clinical trial registration	We did not collect clinical data.
Study protocol	We did not collect clinical data.
Data collection	We did not collect clinical data.
Outcomes	We did not collect clinical data.

ChIP-seq

Data deposition

- [ ] Confirm that both raw and final processed data have been deposited in a public database such as GEO.
- [ ] Confirm that you have deposited or provided access to graph files (e.g. BED files) for the called peaks.

Data access links *May remain private before publication.*	We did not collect ChIP-seq data.
Files in database submission	We did not collect ChIP-seq data.
Genome browser session (e.g. UCSC)	We did not collect ChIP-seq data.

Methodology

Replicates	We did not collect ChIP-seq data.
Sequencing depth	We did not collect ChIP-seq data.
Antibodies	We did not collect ChIP-seq data.

Peak calling parameters	We did not collect ChIP-seq data.
Data quality	We did not collect ChIP-seq data.
Software	We did not collect ChIP-seq data.

Flow Cytometry

Plots

Confirm that:

- [ ] The axis labels state the marker and fluorochrome used (e.g. CD4-FITC).
- [ ] The axis scales are clearly visible. Include numbers along axes only for bottom left plot of group (a 'group' is an analysis of identical markers).
- [ ] All plots are contour plots with outliers or pseudocolor plots.
- [ ] A numerical value for number of cells or percentage (with statistics) is provided.

Methodology

Sample preparation	We did not use flow cytometry.
Instrument	We did not use flow cytometry.
Software	We did not use flow cytometry.
Cell population abundance	We did not use flow cytometry.
Gating strategy	We did not use flow cytometry.

- [ ] Tick this box to confirm that a figure exemplifying the gating strategy is provided in the Supplementary Information.

Magnetic resonance imaging

Experimental design

Design type	We did not use magnetic resonance imaging.
Design specifications	We did not use magnetic resonance imaging.
Behavioral performance measures	We did not use magnetic resonance imaging.

Acquisition

Imaging type(s)	We did not use magnetic resonance imaging.
Field strength	We did not use magnetic resonance imaging.
Sequence & imaging parameters	We did not use magnetic resonance imaging.
Area of acquisition	We did not use magnetic resonance imaging.

Diffusion MRI ☐ Used ☐ Not used

Preprocessing

Preprocessing software	We did not use magnetic resonance imaging.
Normalization	We did not use magnetic resonance imaging.
Normalization template	We did not use magnetic resonance imaging.
Noise and artifact removal	We did not use magnetic resonance imaging.
Volume censoring	We did not use magnetic resonance imaging.

Statistical modeling & inference

Model type and settings: We did not use magnetic resonance imaging.

Effect(s) tested: We did not use magnetic resonance imaging.

Specify type of analysis: ☐ Whole brain ☐ ROI-based ☐ Both

Statistic type for inference (See Eklund et al. 2016): We did not use magnetic resonance imaging.

Correction: We did not use magnetic resonance imaging.

Models & analysis

n/a	Involved in the study
☐	☐ Functional and/or effective connectivity
☐	☐ Graph analysis
☐	☐ Multivariate modeling or predictive analysis

Functional and/or effective connectivity: We did not use magnetic resonance imaging.

Graph analysis: We did not use magnetic resonance imaging.

Multivariate modeling and predictive analysis: We did not use magnetic resonance imaging.
